# LSGI: interpretable spatial gradient analysis for spatial transcriptomics data

**DOI:** 10.1186/s13059-025-03716-1

**Published:** 2025-08-08

**Authors:** Qingnan Liang, Luisa Solis Soto, Cara Haymaker, Ken Chen

**Affiliations:** 1 Department of Bioinformatics and Computational Biology, UT MD Anderson Cancer Center, 7007 Bertner Avenue, Houston, TX 77030 USA; 2 Department of Translational Molecular Pathology, UT MD Anderson Cancer Center, Houston, USA

## Abstract

Cellular anatomy and signaling vary across niches, which can induce gradated gene expressions in subpopulations of cells. Such spatial transcriptomic gradient (STG) makes a significant source of intra-tumor heterogeneity. We present *Local Spatial Gradient Inference* (LSGI), a computational framework that systematically identifies spatial locations with prominent, interpretable STGs from spatial transcriptomic (ST) data. We demonstrate LSGI in tumor ST datasets and identify pan-cancer and tumor-type specific pathways with gradated patterns, highlighting the ones related to spatial transcriptional intratumoral heterogeneity. LSGI enables interpretable STG analysis, which can reveal novel insights in tumor biology from the increasingly reported tumor ST datasets.

**Supplementary Information:**

The online version contains supplementary material available at 10.1186/s13059-025-03716-1.

## Background

Tumor tissues contain heterogeneous cell populations with distinct transcriptional, genetic, and epigenetic features in a complex cellular microenvironment [1–3]. Dissecting such multifactorial intratumoral heterogeneity (ITH) is fundamental to understanding tumor initiation, metastasis, and therapeutic resistance [4–10]. One source of transcriptional variation in cells is their microenvironments, which shape gene expression through different ways, such as cell–cell communication (e.g., ligand-receptor signaling) or local signaling cues (e.g., pH, oxygen, metabolites). As a result, some cells would display gradated transcriptional variation along with their spatial localizations, therein termed “spatial transcriptomic gradient” (STG). Identification of STGs can greatly enhance our understanding of the spatial-phenotypic relationship of cells, enhancing the discovery of multicellular signaling [11] that is elusive in current cell-type-centric investigations. For instance, the oxygen gradient has been shown to shape intra-tumoral heterogeneity affecting tumor proliferation in over 19 tumor types [12–14].

The development of spatial transcriptomics (ST) technologies [15–17] allows simultaneous characterization of gene expressions and tissue context of cells in a high-throughput manner, and thus provides sufficient information for systematic identification of STGs in tissue samples. For instance, hallmark pathway gradients have been observed across the tumor-TME boundary in liver cancer slides along the pre-defined axis perpendicular to that boundary [18]. However, there is an unmet analytical need to perform de novo discovery of STGs without prior pathological annotations, and to discover molecular-spatial heterogeneity beyond apparent pathological annotations. To our best knowledge, no method exists that can detect simultaneously the existence and direction of STGs, which can vary abruptly and substantially across neighboring niches. Trajectory inference (TI) approaches [19, 20] developed for single-cell transcriptomic data analysis cannot be readily applied due to their assumptions on continuity.

Here, we report a novel computational framework, LSGI (Local Spatial Gradient Inference), that performs de novo detection, characterization, and visualization of STGs from ST data. LSGI aims at reconstructing salient STGs across spatial niches. As a highly flexible framework, LSGI combines cell phenotype quantification (e.g., pathway activity) with linear models to simultaneously detect the existence and direction of linear spatial gradients in each small niche. It applies NMF to derive quantitative, interpretable cell phenotypes from the gene expression matrix of a ST data. We demonstrated the utility of LSGI in detecting STGs of different cell phenotypes in tumor samples with aberrant cellular composition and tissue reorganization. In particular, we revealed the spatial proximity of different phenotypes, highlighting an opposite-directed gradient of neural progenitor-like phenotype and hypoxia phenotype in the intratumoral region of a glioblastoma sample. In the meantime, we showed the versatility of the LSGI framework in calculating and visualizing spatial gradients in other spatial technologies and in single-gene manners by highlighting gene gradient patterns in mouse spinal cord development using a seqFISH dataset. We further applied LSGI to perform a meta-analysis on 87 publicly available tumor ST datasets from 9 studies. We identified a total of 356 NMF programs associated with STGs and grouped nearly 3/4 of them into 19 meta-programs (MPs). Some of the MPs were shared by multiple tumor types, while others were tumor-type-specific. About 1/4 of the NMF programs were characterized as sample-specific programs, highlighting inter-patient heterogeneity. We further categorized the programs based on their spatial association with tumor regions, normal regions, or boundary regions, and highlighted NFKB-TNFA signaling pathways as recurrent gradated programs associated with spatial ITH in different glioblastoma samples, which has been reported as a mechanism employed by GBM cells to enhance their resistance [21]. All the processed data of this meta-analysis have been made publicly accessible (https://zenodo.org/records/10626940)[22, 23], and we provide R code to assist visualization and interpretation of the phenotypic gradients. Finally, we report LSGI as an open-source R package https://github.com/qingnanl/LSGI [24].

## Results

### Overview of the LSGI framework

**Fig. 1:**
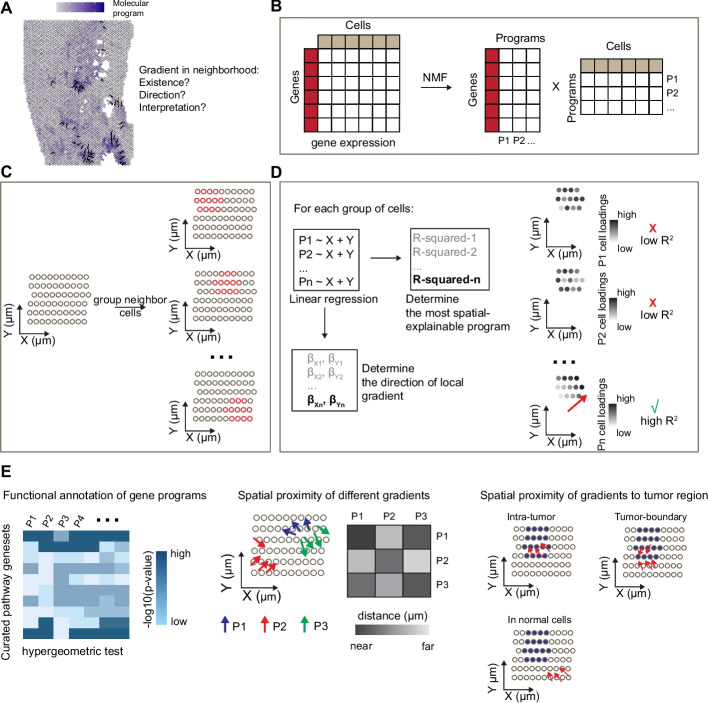
The LSGI framework and downstream analysis. **A** Demonstrative plot showing the existence of a cell phenotypic gradient, summarized by some molecular programs, on a spatial map of tissue. Dark blue color demonstrates higher activity levels of the molecular program. Arrows indicate the direction of gradients. **B** LSGI employs NMF to summarize the gene expression of cells into programs. **C** LSGI partitions cells into small groups based on their spatial localizations. One cell can be assigned to multiple groups. **D** Linear regression is performed in each spatial group of cells by fitting the loading of each NMF program with X and Y coordinate. R-squared is used to evaluate the performance of the regression, while the regression coefficient determines the direction of the local gradient. **E** Downstream analysis on LSGI outputs: functional interpretation of gene programs (left), spatial proximity of different gradients (middle), and spatial proximity of gradients with other biological factors, such as the boundary of the tumor core

The main purpose of LSGI is to characterize spatial transcriptional gradient (STGs) of cells by answering three major questions: first, where does such gradient exist on the spatial map; second, what is the direction of the gradient; and third, what is the functional interpretation of the gradient (Fig. 1A). To achieve this goal, LSGI by default employs NMF to factorize the collective gene expression profiles of all the cells or spots in a ST data into multiple programs (Fig. 1B), including those delineating cellular compositions and those regulating cellular phenotypes. Through this step, cell loadings and gene loadings are calculated, indicating the cell/spot-level activity of the programs and gene-level attribution to the programs, respectively. Since there is no prior information on the locality, linearity, and spatial mode (e.g., simple monotonical gradient or radial-like gradient) of the cells with STGs, we examine the spatial map with a slide-window strategy (Fig. 1C), under which cells are grouped by spatial localizations in overlapping windows (Methods). We then fit linear models using spatial coordinates as predictors and cell NMF loadings as targets for every NMF program and every group of cells (Fig. 1D). *R*-squared is used to evaluate goodness of fit, with larger values indicating the existence of STGs. The direction of a gradient is determined by the corresponding regression coefficients. Provided in the method section is a more comprehensive investigation and analysis of the parameter selection in LSGI, such as the window size, the *R*-squared cutoff, and possible other gene program construction methods.

All these steps described above create a map containing the localization and direction of STGs as well as their assignment to one or more NMF programs. We then functionally annotate the programs by statistical methods (e.g., hypergeometric test) utilizing curated functional gene sets (Fig. 1E, left). We investigate the spatial relationships of gradients assigned to different programs, or the spatial relationship of gradients to tumor-TME boundary in tumor ST datasets (Fig. 1E, middle and right).

### LSGI reveals intratumoral, opposite-directed gradients of cell phenotypes in a GBM dataset

**Fig. 2:**
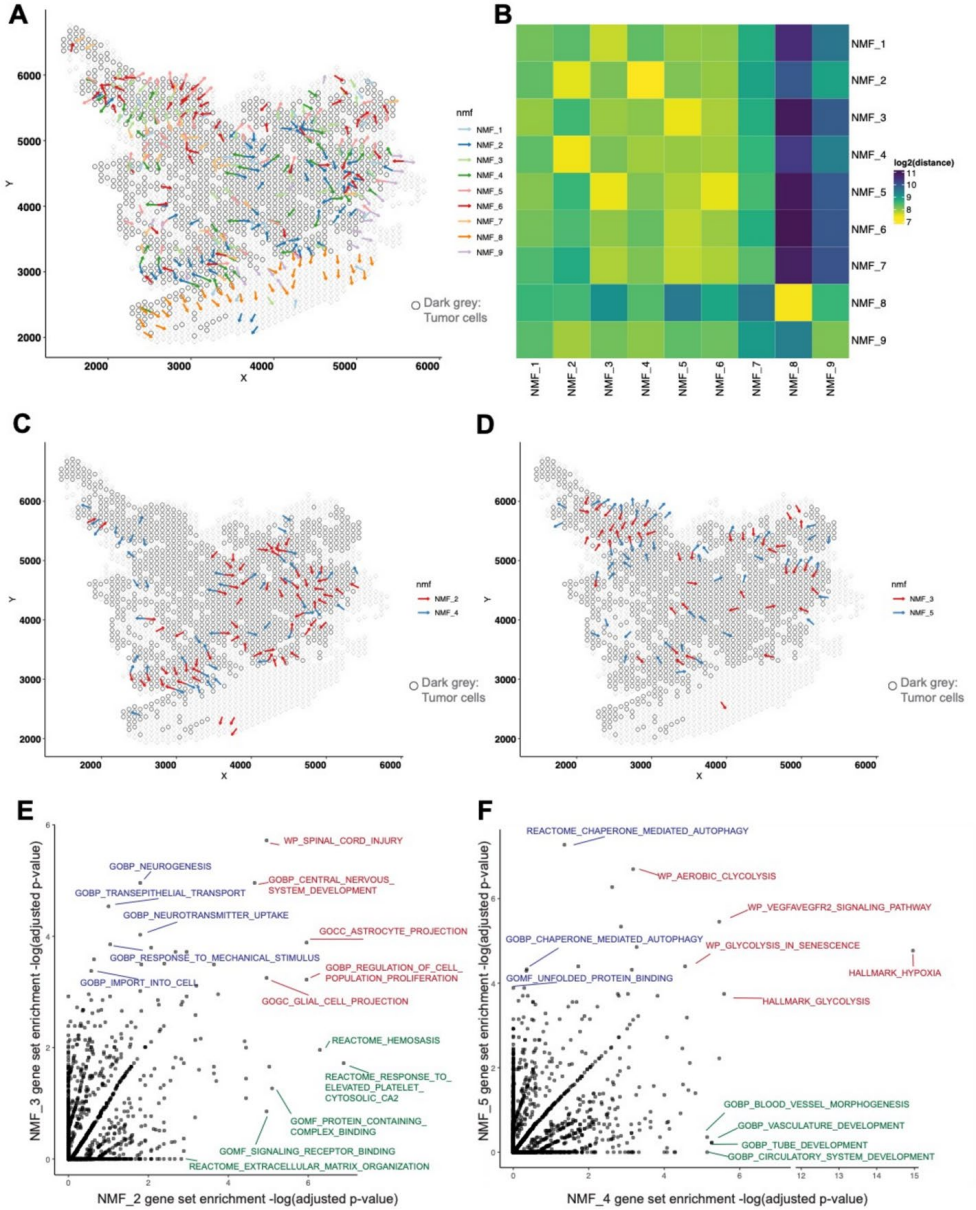
Application of LSGI on single ST dataset. **A** Visualization of LSGI output on the spatial map (dataset: UKF243_T_ST from the RaviGBM study). Each rhombus represents a data spot (10 × Visium technology) while the overlaying dark gray circles represent data spots characterized as tumor regions. Each arrow indicates the presence of a gradient, and the colors represent different NMF programs of this gradient. Arrow directions indicate the direction of gradients. **B** Spatial proximity of different gradients. The colors represent the log-transformed distance from the NMF program in a row to the program in a column. Here the distance is the real physical distance. Notice that this matrix is not symmetric (Methods). **C**–**D** Visualization of the proximal NMF program pairs (C: NMF_2/4; D: NMF_3/5). Each arrow indicates the presence of a gradient, and the colors represent different NMF programs of this gradient. Arrow directions indicate the direction of gradients. The overlaying dark gray circles represent data spots characterized as tumor regions. **E** Comparison of pathway enrichment in top loading genes of NMF_2 and NMF_3. Each data point is a pathway, and the two axes are the -log(adjusted p-value) for the hypergeometric test for enrichment. **F** Comparison of pathway enrichment in top loading genes of NMF_4 and NMF_5. Each data point is a pathway, and the two axes are the -log(adjusted p-value) for the hypergeometric test for enrichment

To investigate the power of LSGI in dissecting tissue heterogeneity, we first applied LSGI to a glioblastoma (GBM) ST dataset [25] (UKF243_T_ST). In this experiment, we empirically identified STGs as those with R-squared higher than 0.6 and visualized them as arrows on the spatial map, colored by their assignment to different NMF programs (Fig. 2A). We also highlighted tumor-harboring spots (through aneuploidy analysis) with gray circles to elucidate the spatial relationship between the STGs and tumor-TME boundaries (Methods, “Cross-sample analysis in 87 tumor ST datasets: preprocessing and tumor region annotation”). We then quantified the mean physical distance between each type of the gradients, through which we noticed that some programs tend to colocalize, such as NMF_2 and NMF_4, or NMF_3 and NMF_5, etc. (Fig. 2B, Methods). Interestingly, we observed that at multiple locations, the NMF_2 and the NMF_4 gradients colocalize yet point towards opposite directions, as if they repel against each other (Fig. 2C, Additional file 1: Fig. S1a–b). Similar patterns were seen among the NMF_3 and NMF_5 gradients (Fig. 2D, Additional file 1: Fig. S1c–d).

To interpret these programs, we performed gene set enrichment analysis for each NMF program (based on top 50 genes by loading levels) through hypergeometric tests (Fig. 2E–F). Interestingly, we found an enrichment of astrocyte and cell proliferation related terms in NMF_2 and NMF_3 (Additional file 1: Fig. S2) and the top genes include *SLC1A3* and *GFAP*, markers of the previously defined “AC-like” tumor cell state [26]. On the other hand, we found an enrichment of hypoxia related terms for NMF_4 and NMF_5 (Additional file 1: Fig. S2) with the top genes *VEGFA*, *NDRG1*, and *ENO1*, markers of previously reported “MES-hypoxia” tumor cell state [27]. Our findings are consistent with a previous study that cells with hypoxia and migration phenotypes display opposite orientations [25]. While the previous findings relied on knowing the genes a priori, our findings were ab initio from the ST data. Besides the shared pathways, we also discovered differentially enriched pathways between each pair. For example, although NMF_2 and NMF_3 both had astrocyte-related terms, NMF_2 had several intercellular interaction terms such as extracellular matrix organization, signaling receptor binding, etc., while NMF_3 programs related to neuron functions such as neurogenesis and neurotransmitter uptake. Similarly, although NMF_4 and NMF_5 both had hypoxia and glycolysis terms, NMF_4 specifically had blood vessel development related terms, while NMF_5 had autophagy related terms, highlighting different reactions upon hypoxia signals. Our findings also imply that different phenotypes marked by the paired NMF_2/3 and the NMF_4/5 programs are functionally coupled to each other.

In the meantime, we applied a recent method, STew [28], which partially shared the scope of finding gradated signals in ST data, to the same GBM dataset. With default parameters, we identified 20 spatially informed canonical vectors (sCVs) and 8 of them showed canonical correlation coefficients larger than 0.5 (Additional file 1: Fig. S3a). The overall sCVs cell embeddings and the NMF cell embeddings showed moderate similarity (RV coefficient 0.33) (Additional file 1: Fig. S3b). Intriguingly, we noticed that some NMF programs and sCVs showed high correlation in their cell loadings, exemplified by sCV5 and NMF2, sCV6 and NMF8, and sCV9 and NMF6 (Additional file 1: Fig. S3c–h). These results support the robustness of both methods, while also elucidating the impact of different information extraction methods on gradated signal detection from ST datasets.

### LSGI discovers and visualizes single-gene gradient in mouse spinal cord development

We sought to explore the versatility of the LSGI framework in analyzing data yielded from other spatial genomics technologies, such as the imaging-based technologies that specify target panels before the experiment. Here we analyzed a seqFISH mouse embryo dataset [29] and focused on the spinal cord region (1237 cells) explicitly (Additional file 1: Fig. S4a). Given the limitation of constructing gene programs from a manually selected gene panel (351 genes), we explored an alternative way of performing LSGI by calculating the gradient of each gene individually. Of all the 351 genes, 60% of them (212) showed gradients in at least 5% of total grids (12 out of 247 grids). We discovered and herein demonstrate some of these genes that show gradated expression patterns, such as *Sox2*, *Shh*, and *Notch1*. Interestingly, *Sox2* showed a trend of higher expression at both the dorsal and ventral sides and lower in the middle (Additional file 1: Fig. S4b), while *Shh*, known for its function of ventral neuron differentiation, showed higher expression in the ventral side only middle (Additional file 1: Fig. S4c). Another key signaling molecule, *Notch1*, showed higher expression in the central spinal cord than either the dorsal or ventral side middle (Additional file 1: Fig. S4d). These visualizations highlighted distinct patterns and dynamics of signaling molecules in a single organ during its development and supported the capability of LSGI in spatial pattern discovery not only in sequencing-based spatial transcriptomics but also in imaging-based datasets, with a gene-specific manner instead of the gene program level. It is worth mentioning that LSGI is highly scalable and efficient when the number of features for gradient test increases (from 10-ish to a few hundred in this case), taking less than 1 min to measure the gradients of all 351 genes (16 Gb RAM, Apple M1 chip). Finally, a tutorial for performing such single-gene gradient analysis is added to our repository [24] of the software.

### Systematic analysis of 87 tumor ST datasets with LSGI

**Fig. 3:**
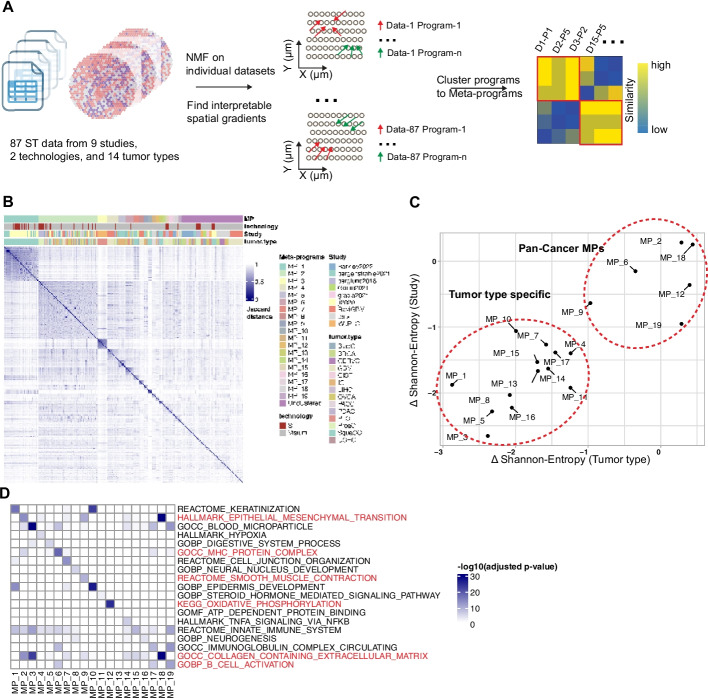
Cross-sample analysis of tumor datasets with LSGI. **A** Schematic of the study design. LSGI was applied to each dataset separately, and the NMFs were then integrated through clustering. **B** Information of the 19 meta-programs. The heatmap showed the Jaccard distance between programs (using top 50 genes). Each program was labeled with the meta-program, technology, study, and cancer type information. **C** Study and tumor type specificity of each meta-program. The theoretical maximum Shannon entropy was calculated for each meta-program based on the tumor type and study label through averaging of random shuffling labels. These entropy quantifications were further subtracted by the real compositional Shannon entropy of the meta-program. **D** Functional annotation of each meta-program. The pan-cancer meta-programs were highlighted with red labels of the annotation term

To perform systematic tumor STG discovery, we further collected 87 ST datasets from 9 different studies (Table 1, Additional file 2: Table S1) including samples from a variety of tumor types. We performed LSGI independently on each sample (Fig. 3A) and obtained at least one gradated NMF program greater than the empirical *R*-squared cutoff (> 0.6) in 75 of the 87 datasets. From these NMF programs, we curated 19 meta-programs (Fig. 3B) after merging similar programs using an approach published previously [30] (Methods, “Clustering NMF programs to meta-programs”). Some meta-programs consist of programs deriving from one tumor type or one study, while others were recurrent (Fig. 3B, Additional file 1: Fig. S5a–b). For each meta-program, we used the delta-Shannon entropy to quantify whether a large fraction of the meta-program originated from a single tumor type or study (Fig. 3C, Methods “Calculation of compositional entropy”). Among the 19 meta-programs, 6 were identified as pan-cancer ones, while the others were cancer type specific. We further annotated the meta-programs using functionally curated gene sets (Methods, Fig. 3D) and visualized the loadings of the genes from assigned pathways in each program, grouped by the meta-program (Additional file 1: Fig. S5c). Of particular interest are the pan-cancer meta-programs related to EMT (epithelial mesenchymal transition), OXPHOS (oxidative phosphorylation), smooth muscle, extracellular matrix, and immune (MHC complex and B cell activation). The functional annotation of cancer-type specific meta-programs also showed consistency with prior knowledge; for example, MP-1 and MP-10 were related to keratinization, and they solely originated from squamous cell carcinoma datasets. Moreover, MP-4 was related to hypoxia and was mostly originated from GBM datasets. Many of the terms have been previously reported in cancer single-cell studies, such as EMT, MHC, hypoxia, neurogenesis, etc. About 1/4 (90 out of 356) the programs were not clustered in meta-programs, highlighting the degree of intra-tumoral heterogeneity. Full information on the factors, their meta-program assignment, and the functional annotation of the meta-programs are reported in Additional file 2: Table S2 and Additional file 3: Table S3.

**Table 1:** A brief summary of the 87 datasets used for cross-sample analysis with LSGI

Study name	Number of datasets	Cancer type(s)
Barkley2022 [31]	10	BRCA (breast cancer), GIST (gastrointestinal stromal tumor), LIHC (liver hepatocellular carcinoma), OVCA (ovarian cancer), PDAC (pancreatic ductal adenocarcinoma), UCEC (uterine corpus endometrial carcinoma)
Bergenstrahle2021 [32]	8	IC (intestine cancer), SquaCC (squamous cell carcinoma)
Berglund2018 [33]	14	ProsC (prostate cancer)
WuPLC [18]	7	PLC (primary liver carcinoma)
Gouin2021 [34]	4	BladC (bladder cancer)
Gracia2021 [35]	4	OVCA
Ji2020 [36]	16	SquaCC
tenx	6	BRCA, CERVC (cervical cancer), IC, OVCA, PACC (prostate cancer, adenocarcinoma with invasive carcinoma), ProsC
RaviGBM [25]	18	GBM (glioblastoma)

**Fig. 4:**
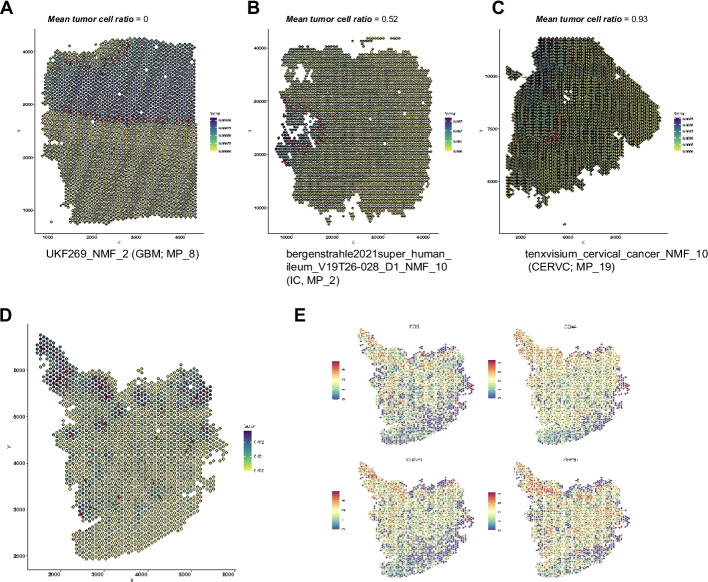
Spatial relationship between gradients and tumor boundary. **A**–**C** Examples of NMF programs with different mean tumor cell ratios. The information of the program, its tumor type, and its meta-program assignment was labeled under each panel. Red arrows marked the presence and direction of the gradient. For each panel, each rhombus represents a data spot while the overlaying dark gray circles represent data spots characterized as tumor regions. The color of the rhombus represents the loading of the NMF program. The labels (e.g. UKF_269_NMF_2) referred to the origins of the datasets, which could be tracked in Supplementary Table_1, and the program (e.g., “NMF_2”) identified from our NMF approaches. **D** The gradient direction and original cell loadings of NMF_6 (UKF243_T_ST) on the spatial map. The overlaying dark gray circles represent data spots characterized as tumor regions. **E** The spatial expression of representative genes in NMF_6 (UKF243_T_ST). Warmer colors (red) indicate higher expression levels

We then sought to investigate whether the spatial locations of the STGs can inform tumor-TME tissue architecture. We performed the analysis in the following steps: First, we annotated tumor spots with aneuploid copy number profiles using CopyKat (Methods, Additional file 1: Fig. S6a). Second, with the tumor/normal spot annotation, we calculated a tumor ratio in each sliding window (Methods, “Calculation of tumor ratios for each local gradient”, Additional file 1: Fig. S6b); Third, for a given STG (associated with one NMF program), we collected the tumor ratio in its constituent sliding windows and calculated the average. Intuitively, a low average tumor ratio indicates that the STG tends to appear within normal tissue regions, while a high ratio indicates that the STG tends to appear within tumoral regions. We demonstrate three examples representing low, medium, and high average tumor ratios, respectively (Fig. 4A–C). In each panel, the overlaying dark gray circles represent data spots characterized as tumor regions by CopyKat (Methods). Indeed, we found lower average tumor ratios indicated association to normal regions while higher values indicated association to tumor regions, and medium values to the tumor-TME boundary. We clustered the average tumor ratios for all the (356) programs and categorized them into three tumor ratio clusters (TRCs, Additional file 1: Fig. S7a–b), and we noticed a differential proportion of TRCs among meta-programs (Additional file 1: Fig. S7c). Here, we demonstrate a few examples of MP_14, annotated as TNFA signaling via NFKB. All four programs in this meta-program were from GBM datasets and three of them (UKF243_T_ST, NMF_6; UKF260_T_ST, NMF6; UKF255_T_ST, NMF_3) were enriched in the intratumoral region (all belonged to TRC Cluster 3). We confirmed their localizations in intratumoral regions through visualization of the gradients and representative genes such as *FOS*, *CD44*, *DUSP1*, and *ZFP36*. (Fig. 4D–E, Additional file 1: Fig. S8). The activation of the TNF-NFKB axis has already been revealed in several tumor types including GBM [21, 37], while here, through a systematic analysis, we unraveled its association with spatial intratumoral heterogeneity, with consistency in several patient samples. Finally, all the LSGI outputs for these tumor datasets were made accessible (https://zenodo.org/records/10626940) [24] and sample codes and detailed tutorials were available to the community to freely explore and visualize the data.

## Discussion

We demonstrated the utility of LSGI on sample datasets generated by 10X Visium and an early version of spatial transcriptomics [38], as they have whole-transcriptomic coverage thus enabling unbiased functional interpretation of NMF programs. The spatial resolution of those technologies, however, can limit the power of discovery and confound the results due to cell admixing in spots. This is a limitation of the data, not of LSGI. The LSGI framework can be applied agnostically to technologies, as the only required inputs are spatial coordinates and gene expression levels. As single-cell resolution whole transcriptomic ST technologies [39] become increasingly available, we expect a relatively straightforward adaptation of LSGI into new technologies. For example, when single-cell resolution whole transcriptomic ST technology becomes more available and yields high-quality datasets, we could combine the results of LSGI and the fine cell type annotation to facilitate the interpretation of LSGI results. We could possibly map and infer the source of the gradient at the cell type or cell state level and achieve better biological relevance from the data. Moreover, we demonstrated that LSGI can be applied in a single-gene manner on datasets with targeted ST technologies through the seqFISH data analysis example. Lastly, although not demonstrated in this study, LSGI can easily fit three-dimensional ST data analysis through adding an additional “Z” coordinate to the linear regression step.

In our work, we mostly focused on the detection, visualization, and analysis of gene program-level gradients, instead of gene-level gradients. We moved in this direction because of two reasons: first, as of the current state of spatial genomics technologies, the sequencing depth of each cell or spot remains limited, thus causing high sparsity in gene expression measurements, and this would potentially hamper the gradient estimation; second, we consider that it is usually a group of functionally coordinated genes, rather than a single gene, that reflects the phenotypical states of cells. With these two reasons stated, we noticed that the gradient of single genes could also be very meaningful, especially for critical genes that decide cell phenotypes, and our computational framework has the flexibility to take the gene expression matrix as the input and calculate single-gene gradients with good scalability. Our example of analyzing the mouse embryo spinal cord data (Additional file 1: Fig. S4) demonstrated such flexibility of LSGI.

Noticeably, several other methods [28, 40, 41] also aimed to detect gradated signals in ST data. While these methods focus largely on inference of global spatiotemporal trends from continuous gene expression data, LSGI focuses on detecting interpretable, phenotypically salient gradients factorizable by NMF. We propose that caution needs to be taken when attempting to use all cells/spots to infer a “global” gradient, because when no biologically meaningful gradients are present (for instance, distinct cell types mixed together in some regions of complex tissues), trajectory inference method may overfit the data. To this end, LSGI benefits from its design that the existence of each local gradient is assessed by how well the local linear model fits the data.In practice, we found that many regions on ST data do not have salient gradients of any programs. In the meta-analysis approach of this study, 12 of the 87 tumor samples had no salient gradient identified. Finally, LSGI is benefited from its utilization of NMF to extract transcriptional phenotypes from the expression matrix because NMF has been shown capable of capturing biological signals [42] and have been widely applied in single-cell or ST studies [30, 31, 43–45]. The employment of NMF not only enhances the interpretability of LSGI but also allows effective cross-sample comparison of the programs, as was previously reported, which laid the basis of our meta-analysis approach to find recurrent STGs in different tumors. Admittedly, although NMF is highly powerful in extracting signals and retain high interpretability, the interpretation of NMF programs in complex biological contexts could still be challenging. In tumors and their microenvironments, gene programs could have complex meanings while precise interpretation of these meanings might require more information than provided by LSGI, such as find cell type annotation. This echoes our point of benefit gained from high resolution spatial transcriptomics data as stated above. Alternatively, instead of NMF, LSGI can allow using pathway activity scores as the input embedding and carefully constructed pathway gene sets may allow better analysis of spatial gradients in such cases. Another limitation of NMF comes from a manually selected number of factors $k$. Although we applied a cross-validation metric to select an optimal $k$ and a reconstruction tolerance value to control for overfitting, it is possible that the $k$ parameter is not precisely biologically relevant. Thus, when analyzing the LSGI results, it is recommended to carefully analyze the interpretation of the factors, as well as leveraging domain knowledge of the complexity of biological signals in the sample whenever possible.

We also applied other dimensionality reduction methods, namely PCA and ICA, on our 87 pan-cancer datasets, and compared the results with the NMF results. In general, we noticed that PCA results showed good consistence with NMF results (most RV coefficients in the range of 0.65–0.9) and were more consistent to NMF results than ICA (Additional file 1: Fig. S9a). We demonstrated this observation with one specific example, that the Pearson’s correlation coefficient between NMF programs and PCA loadings always had a larger absolute value than that of ICA (Additional file 1: Fig. S9b–c). We also showed two examples that NMF loadings allowed for better detection of spatial gradients in the frame of LSGI, as the numerical scale of PCA or ICA loadings could be much larger than that of NMF, thus leading worse fitting of linear models (lower ${R}^{2}$ values) (Additional file 1: Fig. S9d–i). Moreover, with all the gene loadings of NMF being positive, the NMF method is more interpretable in the context of gene programs than PCA or ICA, which explains the wider application of NMF and its variants in the gene program construction field over PCA or ICA. In addition, we investigated whether the detection of gradients with the NMF programs was biased towards regions with higher detection rate (more genes with non-zero values), and we found that although there were several cases showing positive or negative correlations between detection rate and maximum gradients, the overall pattern of such associations does not support the existence of such bias (Additional file 1: Fig. S10). In summary, we believe that LSGI serves as a powerful and complementary approach to the other methods targeting alternative scopes and resolution.

Although by default, LSGI uses basic NMF to factorize gene expression programs, the LSGI framework is flexible and can accommodate variants of NMF methods, such as cNMF [46], iNMF [47], and jNMF [48], or other types of cell phenotypic quantification, such as pathway activity measurements [49, 50] to calculate their spatial gradients. We do recommend using NMF if no assumptions were made, and if the users required a systematic, unbiased analysis. For datasets generated with targeted ST technologies [51, 52], we suggest that users be cautious in annotating the NMF programs as the gene set (panel) may be biased towards some pathways due to biased selection of genes. Studying gradients at the single-gene level would be an alternative strategy, exemplified in our seqFISH analysis.

## Conclusions

In this study, we introduced a simple, flexible yet highly interpretable strategy, LSGI, for discovering spatial transcriptomic gradients in a ST data. Given the uncertainty of the existence and spatial variation of STGs, we employed a divide-and-conquer strategy by calculating local linear gradients in sliding windows, which collectively produce an STG map across the tissue. We demonstrated the utility of LSGI for both in-depth, single dataset analysis and cross-sample meta-analysis using 87 tumor ST datasets. Without any prior knowledge, from merely 87 samples, LSGI was able to identify gene expression programs consistent with prior cancer studies and discover patterns indicative of spatial transcriptional heterogeneity on each tissue slide, providing novel functional annotations and insights that would otherwise be missed by the current ST data analysis practices [53] (cell clustering and annotation, spatial niche assignment, spatially variable gene analysis, etc.), or manual, image-based annotations. Compared to the approaches that summarize spatial data into static spatial domains, we showed that spatial gradient approaches were capable of deconvoluting the tumor state dynamics in the spatial context. As we showed, some tumor regions could be associated with different types of phenotypical gradients at various levels, while the assignment of such regions to a single niche/domain would likely lose such a dynamical view. Overall, the development of LSGI has a strong potential for revealing the uninvestigated associations between STGs and pathological/morphological annotations to deepen our knowledge of molecular pathology, leveraging the increasingly rich spatial toolkits and datasets.

## Methods

### The LSGI framework

The main LSGI framework starts from clustering spots (or cells for datasets with single-cell resolution; we would refer to this unit as cell in the following description for simplicity) into small groups solely based on their localization. The number of groups $P$ is controlled by a parameter$S$, that $P= \frac{N}{S}$, where $N$ is the total number of cells. By default, $S$ is set to 5, while the group size $Q$ is set to 25. Thus, each cell is included in $\frac{Q}{S}=5$ groups on average. These parameters were used for all the analysis in this study unless specifically mentioned. The selection of $Q$ controls the resolution of the gradient detection. Setting a smaller $Q$ would let the LSGI program examine linear gradients within smaller window sizes (higher resolution) while also has the risk of reduced robustness to noise due to smaller sample sizes in multivariate regression. With some of the real-world data we used in this study, we noticed an expected trend of larger $Q$ selections provide higher robustness of the linear regression’s performance against false-positives, while a $Q$ selection as low as 10 would detect false-positive gradients (from shuffled spatial localizations) with medium-levels of ${R}^{2}$ (Additional file 1: Fig. S11a-c). Shuffling spatial localizations were supposed to erase all spatial signals, while at $Q$=10 the shuffled group still could call a relatively large proportion of gradients as positive with ${R}^{2}$ larger than 0.4. When the $Q$ increase to 25, the shuffled group hardly retain any called gradients with ${R}^{2}$ larger than 0.4.

We also require such groups of cells to be tiling for reducing unwanted effects of arbitrarily determining the groups by suggesting a smaller $S$ than $Q$. To achieve such tiling, we used the balanced_clustering function in the “anticlust” R package [54] to cluster cells into $P$ groups based on the spatial coordinates and determine a grid point at the center of each cluster. We then search for the $Q$ nearest neighbors among cells to each grid point, based on Euclidian distance, thus forming the groups.

By default, LSGI take NMF embeddings of cells as the input. The NMF step is not incorporated in the LSGI framework as many NMF implementation have been reported and we want to offer this flexibility to users. All the NMF step involved in this work used the NMF implementation of the singlet R package [55]. With the group and NMF information, a linear regression is performed for each NMF program in each group: ${F}_{ij} \sim {X}_{i}+ {Y}_{i}$. ${F}_{ij}$ is the loading of the cells from the $i$ th group of the $j$ th NMF program. ${X}_{i}$ and ${Y}_{i}$ are the spatial coordinates of the $i$ th group of the cells. The regression coefficients ${\beta }_{Xij}$ and ${\beta }_{Yij}$ determine the most likely gradient direction of this program $j$ in the group $i$, while the ${R}^{2}$ of this regression represents the largest explanatory capability of spatial effects on the cell loadings of program $j$. Such processes are performed iteratively for all NMF programs and all cell groups. Although ${R}^{2}$ has a clear statistical meaning, the selection of its threshold could be empirical given different contexts. In this study, we only treated the cases where ${R}^{2}\ge 0.6$ as valid gradients and these were retained for further analysis unless otherwise specified. As ${R}^{2}$ equals to 0.5 often treated as a moderate goodness of fit and our rationale was to call the gradient where a slightly higher proportion of the molecular signal (NMF loadings) explained by the spatial localization. In our study of the 87 tumor spatial datasets, setting an ${R}^{2}$ cutoff at 0.6 retains top 1–10% of total possible gradients ($\#NMF\;programs\times\#grids$) (Supplementary Fig. 11A–C). Similarly, with a simulation study, we demonstrate that for well-organized spatial spots (such as those of 10 × Visium), ${R}^{2}$ in a range of 0.4 to 0.6 would distinguish true gradated signals from random noise signals, for $Q$ parameter selections equal to 25 or larger, while this ${R}^{2}$ would not be sufficient to reject false positives when $Q$ is as low as 10 (Additional file 1: Fig. S12).

Besides the value of ${R}^{2}$, we only retain programs with gradients in at least 5% of total grid points, by default. As for the “arrow” visualization (such as Fig. 2A), the arrow directions are pointing to increased program signals (such as Fig. 4D). Please note that it is possible that one group of cells can have different gradients assigned to different NMF programs (usually gradated to different directions).

Furthermore, the LSGI package offers a strategy to estimate the overall distance between two types of gradients (as is shown in Fig. 2B):

Overall distance from a gradient $A$ to gradient $B$, $D\left({F}_{A}, {F}_{B}\right)$, is calculated:

$$D\left({F}_{A}, {F}_{B}\right)=mean\left({d}_{A1}^{B}, \dots , {d}_{Ai}^{B}\right), i=1, 2, 3, \dots , K$$

  

$${d}_{Ai}^{B}=\text{min}\left(distance\left({G}_{Ai}, {G}_{B1}\right), ..., distance({G}_{Ai}, {G}_{Bj})\right), j=1, 2, 3, \dots , L$$

K is the number of grid points ($G$) with gradated program $A$, L is the number of grid points with gradated program $B$, $distance$ here is Euclidean distance. In short, for each grid point $i$ with program $A$ (${G}_{Ai}$), we find the closest grid point with program $B$ and record that distance (${d}_{Ai}^{B}$). We then use the mean of this distance of all grids with program $A$ as an overall evaluation of closeness from gradient $A$ to gradient $B$. Please note here $D\left({F}_{A}, {F}_{B}\right) \ne D\left({F}_{B}, {F}_{A}\right)$.

LSGI is an efficient program that the main gradient inference step takes less than 1 min for each dataset in our practice (roughly 3000–8000 spots per dataset, 16 GB RAM MacOS laptop). The LSGI R package has been tested on MacOS (Ventura 13.6), Windows (Windows 11), and Linux (Redhat Enterprise) systems.

### Analyzing the seqFISH data

The seqFISH data [29] was downloaded from (https://marionilab.cruk.cam.ac.uk/SpatialMouseAtlas/) and we analyzed the “embryo1” of the whole dataset. We directly followed the cell type annotation conducted by the authors and provided in the metadata. To perform LSGI analysis, we used the raw counts matrix instead of the NMF loadings as the input of argument “embeddings” when running the function “local.traj.preprocessing” of LSGI. Given the smaller number of overall cells and the higher sparsity of gene counts than NMF loadings, we used window size ($Q$ parameter) equal to 20 and the ${R}^{2}$ cutoff equal to 0.3.

### Cross-sample analysis in 87 tumor ST datasets: preprocessing and tumor region annotation

All the ST datasets were curated and converted to Seurat objects and were preprocessed following the SeuratV4 [56] workflow, including normalization, scaling, dimensionality reduction (with PCA) and clustering, with default parameters. NMF was performed with the singlet R package [55], scanning the number of factors $k$ with a range from 6 to 10. The final $k$ value was decided by cross-validation implemented in the same package. One other criterion for $k$ value was the reconstruction tolerance of NMF decomposition. We followed the default setting of this tolerance equal to 1e − 5 in the Singlet R package. This would avoid overfitting when the data was not complex enough and may yield NMF results with gene programs fewer than 6. Tumor regions of ST datasets were inferred using CopyKat [57] with automatically determined normal cell references. Given the prevalence of immune cells in tumor samples, we sought to use immune cells as the normal cell references (Supplementary Fig.  6 A). We quantified the expression of a set of immune related genes (CD3E, CD8A, GZMK, CD4, CCR7, GZMB, FCER1G, LHDB, DUSP2, IL7R, S100A4) at the single-cell level, and then treat the cluster (from Seurat) with the most top immune-related cells (top 100 cells with highest immune gene expression) as the normal cell reference. The other parameters were default for CopyKat.

### Calculation of tumor ratios for each local gradient

With the annotation of tumor spots, we could obtain the tumor ratio for each grid point (Additional file 1: Fig. S6). For a gradated NMF program of a dataset, we collected the tumor ratio for all grid points where it showed gradient (${R}^{2}\ge 0.6$) and used the average tumor ratio to concisely summarize the spatial relationship between that program and tumor core, normal tissue, or tumor-TME boundary. To cluster programs into different tumor ratio clusters (TRCs), equal-weighted one-dimensional K-nearest neighbors clustering were applied (Additional file 1: Fig. S7).

### Clustering NMF programs to meta-programs

We then applied LSGI separately on each dataset and combined the output for an integrative analysis. We retained only the gradated NMF programs in at least 5% of the total grid points for each dataset. We then clustered the remaining NMF programs following a previously reported approach [30]. Briefly, each cluster of the programs started from a founder program that had the most high-overlapping cases (over 20 overlapping genes among top 50 with highest loadings) with other programs (at least two other programs). The founder program would then be clustered with the program with the highest overlapping genes (and at least 20 overlapping genes), and this meta-program would be assigned a 50-gene signature based on their appearance in the top 50 of each program and their loadings of the original NMF program. The cluster would further grow iteratively following such rules until no programs could be merged into it. Such processes would then start again in the rest of the programs until no founder programs could be identified, and the program left would be assigned to the “Unclustered” group. Thus, each meta-program (except the “Unclustered”) was summarized as a 50-gene signature which facilitated the functional annotation of the meta-program.

### Calculation of compositional entropy

To quantify whether a meta-program was formed by programs from specific study or tumor types, we calculated the delta-Shannon entropy for each meta-program. The Shannon entropy for each program is: $-\sum_{i=1}^{C}p\left({x}_{i}\right)\text{log}[p\left({x}_{i}\right)]$. $C$ is the number of categories (tumor type or study), while $p\left({x}_{i}\right)$ here is the fraction of meta-program originated from the $i$ th category. We then shuffled the category labels randomly and calculated the simulated random entropy and subtracted the average random entropy (10 times simulation) from the real entropy to obtain delta-Shannon entropy. Such measurement reflect how likely a meta-program is composed of programs from different categories with the same probability.

### Functional annotation of NMF programs and MPs

For functional annotation of NMF programs, we tested the enrichment of functional gene sets in the top 50 genes in each program with highest loadings, while for meta-programs, the 50-gene signatures were directly used. The hypergeometric tests were performed with the R package hypeR [58]. Several functional gene sets were combined as the input: Gene Ontology [59] (Biological Process, Molecular Function, and Cellular Component), MSigDB Hallmarks [60], and Canonical Pathways from MSigDB C2 collection [61]. To decide the annotation of meta-programs, we first reduce the hypergeometric test results to top 40 gene set for each meta-program based on adjusted *p*-value (false discover rate adjusted), and further reduce the result to top 5 based on cross-program specificity. The specificity score for gene set $i$ of meta-program $p$ is calculated by ${E}_{ip}-(\sum_{i\ne p}^{n}{E}_{i})/\left(n-1\right)$. Here ${E}_{ip}$ is the negative log-transformed adjusted *p*-value for gene set $i$ enrichment of meta-program $p$ (hypergeometric test). Full functional annotation results are available in Supplementary Table 3.

### Performing STew, PCA, and ICA analysis

We followed the STew [28] default parameters for all the analysis steps, including preprocessing, variable gene selection, and the core CCA-related functions. PCA and ICA were both calculated with highly variable features (top 2000) selected by the Seurat package (V5 [62]). The overall cell loading association between PCA and NMF or ICA and NMF was assessed by calculating the RV coefficient [63] (FactoMineR R package).

### Simulating spatial patterns with SRTSim

We followed the tutorial of SRTSim [64] and manually created spatial patterns. We simulated 400 genes with true spatial patterns with gradients, while 2000 were randomly distributed in the spatial map. The gene expression simulation has no dropout, expression mean equal to 1, and dispersion equal to 0.5. We created 30 gradated gene lists and 30 random gene lists and calculated their pathway scores among cells. We then used the pathway scores for each cell of these 60 gene lists as the input of LSGI and calculated the gradient level. The pathway scores were estimated using AUCell [65].

## Supplementary Information

Additional file 1: Supplementary Figures. Fig. S1-12.
Additional file 2: Supplementary Tables. Table S1-2.
Additional file 3: Supplementary Tables. Table S3.
Additional file 4. Review history.

## Data Availability

A summary of ST datasets is included in Supplementary Table 1. Most of the datasets were downloaded from the SODB [66] curation (Barkley2022 [31], Bergenstrahle2021 [32], Berglund2018 [33], Gouin2021 [34], Gracia2021 [35], Ji2020 [36]). 10 × datasets were downloaded from the 10 × Genomics website. WuPLC [18] datasets were downloaded from https://ngdc.cncb.ac.cn/gsa-human/browse/HRA000437 [67]. RaviGBM [25] datasets were downloaded from https://datadryad.org/stash/dataset/doi:10.5061/dryad.h70rxwdmj [68]. The LSGI processed data (87 tumor datasets) are available in https://zenodo.org/records/10626940[23]. Sample analysis code (https://zenodo.org/records/10626940/files/LSGI-annotation-and-visualization-demo.html?download=1) are available for users to visualize and explore the data.
