# Supplementary material for: LSGI: interpretable spatial gradient analysis for spatial transcriptomics data
Source: Genome Biol. 2025 Aug 8;26:238. doi: 10.1186/s13059-025-03716-1 (PMC12333196; doi:10.1186/s13059-025-03716-1)
Supplement: Supplementary file 1 — Additional file 1: Supplementary Figures. Fig. S1-12. Additional file 2: Supplementary Tables. Table S1-2. Additional file 3: Supplementary Tables. Table S3. Additional file 4. Review history. [file 13059_2025_3716_MOESM1_ESM.docx]

**LSGI: interpretable spatial Gradient analysis for spatial transcriptomics data**

Qingnan Liang^1^, Luisa Solis Soto^2^, Cara Haymaker^2^, Ken Chen^1^

1. Department of Bioinformatics and Computational Biology, UT MD Anderson Cancer Center.

2. Department of Translational Molecular Pathology, UT MD Anderson Cancer Center.

Corresponding author: Ken Chen, Department of Bioinformatics and Computational Biology, UT MD Anderson Cancer Center, 7007 Bertner Avenue, Houston, Texas, United States, 77030. E-mail: [kchen3@mdanderson.org](mailto:kchen3@mdanderson.org)

Additional File 1: Figs S1-12

Fig S1

A-D. Demonstration of the gradient direction and original cell loadings of NMF_2 (A), NMF_4 (B), NMF_3 (C), and NMF_5 (D) on the spatial map. The overlaying dark grey circles represent data spots characterized as tumor region

Fig S2

A-D. Functional enrichment of top genes in NMF_2 (A), NMF_4 (B), NMF_3 (C), and NMF_5 (D). Bar-plots showed the ratio of pathway genes found in input gene sets (top 50 genes in each NMF program) and were colored by the adjusted p-value (false discovery rate, FDR) of hypergeometric test.

Fig S3

A. The spatial correlation of the 20 STew spatially informed canonical vectors (sCVs) in UKF243_T_ST from the RaviGBM study.

B. Pearson’s correlation coefficients between STew sCV loadings and NMF cell loadings.

C-H. Visualization of compatible STew sCV loadings and NMF cell loadings between sCV5 and NMF2 (C, F), sCV6 and NMF8 (D, G), and sCV9 and NMF6 (E, H).

Fig S4

A. The mouse embryo spatial localization of cells highlighting the spinal cord region.

B-D. Spatial gradient visualization for developmental related genes Sox2 (B), Shh (C), and Notch1 (D) on the mouse embryo spinal cord spatial map. The X and Y axis units are pixels.

Fig S5 Information of meta-program composition and functional annotations.

A-B. Proportion of originated tumor types and study of NMF programs in each meta-program.

C. Loadings of the assigned functional gene set members in each NMF program grouped by meta-program.

Fig S6 Strategy of automatically detection normal cell references and calculate tumor cell ratio in each local group.

A. Strategy to infer immune cell clusters for annotating tumor regions with CopyKat.

B. Calculation of groupwise tumor cell ratio. Red circles represent cells in a local group while grey circles are other cells. Dark blue labels tumor cells. For each group, the tumor ratio equals to the number of tumor cells in this group divided by the number of all cells in the group.

Fig S7 Cluster programs based on tumor spot ratios.

A. Using one-dimensional equal-weighted KNN clustering of tumor cell ratios to form three TRCs.

B. The distribution of tumor cell ratios among programs (right). Colors indicate the identity of three TRCs.

C. Proportion of programs clustered to the three TRCs in each meta-program and the ‘unclustered’ programs.

Fig S8

A. The gradient direction and original cell loadings of NMF_3 (UKF55_T_ST) on the spatial map. The overlaying dark grey circles represent data spots characterized as tumor region.

B. The spatial expression of representative genes in NMF_3 (UKF255_T_ST). Warmer colors (red) indicate higher expression levels.

C. The gradient direction and original cell loadings of NMF_6 (UKF260_T_ST) on the spatial map. The overlaying dark grey circles represent data spots characterized as tumor region.

D. The spatial expression of representative genes in NMF_6 (UKF260_T_ST). Warmer colors (red) indicate higher expression levels.

Fig S9

A. RV coefficients between PCA and NMF compared to that between ICA and NMF, in all 87 pan-cancer ST datasets. Red dashed line showed slope equal to 1.

B-C. Pearson’s correlation coefficients between PCA loadings and NMF cell loadings (B) and between ICA loadings and NMF cell loadings (C) in example dataset Barkley2022Cancer_GIST2.

D-I. Spatial gradients for NMF_4 (D), PC_2 (E), IC_10 (F), NMF_6 (G), PC_1 (H), and IC_8 (I) detected by LSGI. For better visualization, PC_1 (H) was visualized using the additive inverse of its original cell loadings.

Fig S10

Correlation between detection rate of spatial windows and the gradient in 87 cancer ST datasets.

A. Histogram of the Pearson’s correlation coefficients between the detection rate of spatial windows and the maximum $R^{2}$ of the gradients detected in that window.

B-D. Examples of the correlations between maximum gradient and detection rates in three scenarios: positive (B), negative (C), and neutral.

Fig S11

A-C. Spatial gradient detection with different $Q$ parameter (window size) and $R^{2}$ cutoff in raw and shuffled real-world data. Three demonstrative datasets in A-C as labeled. Different window sizes were tested from 10 to 200, and the heatmap color depicts the proportion of total possible gradients called with given $R^{2}$ cutoffs. Here, ‘gt_0.1’ means cutoff equals to 0.1, etc. The red dashed boxes highlight the parameter selected with this study.

Fig S12 Analyzing $R^{2}$ cutoffs with simulation data.

A-B. Demonstration of simulated spatial signals and random signals by SRTSim.

C-E. Calling true spatial gradient signals and random signals at different window size ($Q$): 10 (C), 25 (D), and 50 (E). The cutoff_1 to cutoff_9 refer to the scenario of setting $R^{2}$ cutoffs equal to 0.1 to 0.9.
